# Assessing the Mental Health of Emerging Adults Through a Mental Health App: Protocol for a Prospective Pilot Study

**DOI:** 10.2196/25775

**Published:** 2021-03-02

**Authors:** Asal Yunusova, Jocelyn Lai, Alexander P Rivera, Sirui Hu, Sina Labbaf, Amir M Rahmani, Nikil Dutt, Ramesh C Jain, Jessica L Borelli

**Affiliations:** 1 Department of Psychological Science University of California, Irvine Irvine, CA United States; 2 Department of Economics University of California, Irvine Irvine, CA United States; 3 Department of Computer Science University of California, Irvine Irvine, CA United States; 4 School of Nursing University of California, Irvine Irvine, CA United States; 5 Department of Electrical Engineering and Computer Science University of California, Irvine Irvine, CA United States; 6 Department of Cognitive Sciences University of California, Irvine Irvine, CA United States

**Keywords:** ecological momentary assessment, stress, digital mental health, college student, mental health, protocol, prospective, feasibility, individual, factors, sleepy, physiology, activity, COVID-19

## Abstract

**Background:**

Individuals can experience different manifestations of the same psychological disorder. This underscores the need for a personalized model approach in the study of psychopathology. Emerging adulthood is a developmental phase wherein individuals are especially vulnerable to psychopathology. Given their exposure to repeated stressors and disruptions in routine, the emerging adult population is worthy of investigation.

**Objective:**

In our prospective study, we aim to conduct multimodal assessments to determine the feasibility of an individualized approach for understanding the contextual factors of changes in daily affect, sleep, physiology, and activity. In other words, we aim to use event mining to predict changes in mental health.

**Methods:**

We expect to have a final sample size of 20 participants. Recruited participants will be monitored for a period of time (ie, between 3 and 12 months). Participants will download the Personicle app on their smartphone to track their activities (eg, home events and cycling). They will also be given wearable sensor devices (ie, devices that monitor sleep, physiology, and physical activity), which are to be worn continuously. Participants will be asked to report on their daily moods and provide open-ended text responses on a weekly basis. Participants will be given a battery of questionnaires every 3 months.

**Results:**

Our study has been approved by an institutional review board. The study is currently in the data collection phase. Due to the COVID-19 pandemic, the study was adjusted to allow for remote data collection and COVID-19–related stress assessments.

**Conclusions:**

Our study will help advance research on individualized approaches to understanding health and well-being through multimodal systems. Our study will also demonstrate the benefit of using individualized approaches to study interrelations among stress, social relationships, technology, and mental health.

**International Registered Report Identifier (IRRID):**

DERR1-10.2196/25775

## Introduction

### Background

Chronic stress is associated with a person’s physical and emotional well-being. In the United States, 4.7%-11.2% of adults regularly experience worry, anxiety, nervousness, or depression [1]. Stress is a major factor that may contribute to cardiovascular diseases (eg, stroke) [2,3], and repeated stress exposure is linked to adverse mental health outcomes and behaviors, such as depression, anxiety, self-harm, suicidality, and addiction [4,5]. Adolescence and young adulthood are at-risk periods of development wherein mental disorder and mortality incidence rates largely increase [6-9]. These changes in mental health may, in part, be due to rapid shifts in physical and psychological development during brain maturation [10-12]. Indeed, emerging adults, including college students, experience chronic stress; one-fifth of students meet the criteria for severe behavioral problems [13], approximately 30% of college students meet the criteria for depression [14], and 10% of students screen positively for anxiety disorder [15]. Students often experience increases in allostatic load due to continuous exams, the increased number of nonacademic responsibilities (eg, jobs), changes in social support (eg, moving away from home), social stressors (eg, making new friends), and other events that are associated with living in a new environment. These stressors are also accompanied by uncertainty and challenges to individuals’ identities [16,17]. Given these academic, social, and psychological stressors, it is hardly surprising that universities struggle to meet the demand for on-campus mental health services [18]. Additionally, students who need mental health services the most may not take advantage of these services because of social stigma and pragmatic reasons (eg, time constraints) [18,19]. Therefore, stress reduction and management are crucial for this population, as they may experience high-intensity negative emotions [20]. Furthermore, emerging adults have yet to develop the maturity required for exerting top-down control over intense emotional experiences [21]. The goal of this paper is to introduce a research protocol for a prospective study that examines the feasibility of a multimodal approach to understanding the unique individual nuances of mental health.

Psychologists and human behavior researchers have long understood the importance of adaptive stress responses and emotional functioning in well-being. Understanding the intricacies of mental health and its associations with numerous physical and life behaviors is important for choosing interventions and approaches that promote good mental health. From this perspective, mental health is viewed along a health continuum, wherein individuals may fluctuate across a spectrum of diminishing and flourishing mental health [22]. Psychological functioning is closely associated with physical functioning; the manifestations of mental states may be apparent in a person’s physical state [23]. For example, heart-rate variability (ie, a measure of the variation of time between heartbeats) is linked with the stress responses of individuals with affective disorders [24]. Furthermore, with regard to physiological signs and their relevance to mental health, sleep and physical activity are important factors that are associated with mental health [25,26]. Studies often examine these factors separately; sleep researchers may not account for physiological activation or activity, and physiology experts may not include measures of sleep in their studies. A crucial next step in the field of mental health research is examining the interconnectedness of these factors in real time to increase the ecological validity of mental health assessments. This can be done by taking advantage of advancements in technology that ultimately improve mental health treatments. Recent clinical studies have noted the utility of individualized approaches that address mental health concerns.

For several decades, research has focused on how different strategies for coping with stress or maladaptive emotional experiences (ie, feeling emotions too intensely, feeling emotions for too long, or feeling emotions in the wrong context) [27,28] relate to psychopathology and worsen health [29,30]. Typically, stress management for emotion and mood-related disorders include evidence-based treatments such as cognitive behavior therapy, acceptance and commitment therapy, and dialectical behavior therapy [31-33]. However, clinical researchers have suggested that more personalized models and approaches for understanding individual differences and individuals’ unique experiences may inform research on the risk of developing psychopathology [34]. Recently, clinical research has focused on understanding the complexities of mental health symptoms within and around an individual [35]. Furthermore, clinical researchers have been increasingly using transdiagnostic and precision medicine approaches instead of relying on the typical clinical categories and diagnoses in the Diagnostic and Statistical Manual (DSM) [36-39]. This gradual shift from using the DSM is partially due to inconsistencies in clinical diagnoses (ie, symptoms of different disorders often overlap). More specifically, patients do not always exhibit the same symptoms for the same disorder. For instance, depression might impact an individual’s sleep, but depression might manifest in the form of anhedonia or social withdrawal for other individuals. Furthermore, other clinical diagnoses, such as posttraumatic stress disorder, may result from differing types of trauma, which affect the type of symptoms that an individual might exhibit [40]. In addition, people with different diagnoses (eg, anxiety and depression) share many common features (eg, avoidance and withdrawal) and often benefit from the same or similar interventions (eg, exposure or behavioral activation). The recognition of heterogeneity in symptoms and clinical presentations within diagnostic categories, and the recognition of homogeneity across clinical groups has led many to question the utility of the DSM [37,39]. With the ongoing shift in clinical research, researchers have begun to use individualistic approaches for understanding the risks and development of psychopathology. Researchers have also questioned whether a personalized model of treatment that is based on the unique symptomatology of an individual would result in a more effective means of recovery. Furthermore, it is crucial to examine changes in symptoms and behavior over time.

Clinical researchers have begun to use personalized model approaches that take advantage of the advancement and use of wearable and mobile technology, which can be used to predict and prevent adverse mental health outcomes. Noninvasive wearable devices allow researchers to track features that are relevant to mental health, such as mood, sleep, and physiology [41-44]. The use of intensive, longitudinal approaches (eg, daily reporting and the use of wearables) allows researchers to better understand the manifestations of psychopathologies and predict symptomatology [45-47]. For example, studies have combined subjective reporting for evaluating mood with objective measures for physical activity to understand the associations between negative mood and physical activity [48]. Moreover, researchers have recommended the use of individualized approaches for understanding psychopathology; treatments can be tailored to each individual, as a person’s symptoms may differ from those of another person with the same psychopathology [34]. The Internet of Things (IoT) is a nascent, but rapidly growing paradigm wherein the objects of everyday life are equipped with sensing, processing, storage, communication, and networking capabilities that allow objects to communicate with each other and with users. These objects have become an integral part of the internet [49,50]. In addition, wearable devices (ie, smart wristbands, rings, clothing, etc) form a rapidly emerging new class of IoT technologies named wearable IoT (WIoT) technologies, which have the ability to sense critical physiological, behavioral, and contextual data. WIoT technologies can also analyze, store, and transmit these valuable data [51]. An artificial intelligence–enabled event mining system that operates on such rich big data can be used to assess temporal associations among events, for the purpose of building personalized models. These personalized models can be used to enhance the health and well-being of individuals. A personalized model approach allows researchers to conduct root cause analyses and study interrelations among stress, social relationships, technology, and mental health. To gain a holistic perspective of well-being and factors that contribute to fluctuations in one’s mental health, researchers often use IoT technologies to monitor physical health (ie, sleep, physical activity, and physiology) and behaviors, and to conduct ecological assessments (eg, daily diaries and surveys) for assessing psychological well-being (ie, mood, emotion, and depression). An advantage of a holistic approach includes the ability to identify various environmental and social factors that may be overlooked during standardized diagnostic tests, since it is well known that psychological disorders do not have one root cause [52-54]. Although there are many benefits to using these approaches, a large portion of related literature has only focused on the theoretical advantages [55]. Many studies have yet to examine data that support these theoretical advantages. Indeed, holistic and personalized approaches are a recent, emerging topic in the field of psychology; researchers have used machine learning and network analysis techniques for analyzing intensive, self-reported assessment and wearable data, to examine symptom clusters for depression [56]. Furthermore, this approach may help with informing mental health interventions and health care providers’ clinical recommendations. Studies on the IoT and the use of wearables in health monitoring have suggested that clinicians may be able to use WIoT technology–based information to complement their diagnoses and recommendations [57]. The recent advancements in WIoT technology research have allowed researchers to use personalized and holistic approaches for understanding the development of mood disorders.

### Objective

In this protocol paper, we describe a prospective study that aims to assess the effectiveness of a multimodal approach for establishing a more comprehensive understanding of an individual’s experience. We will achieve this by conducting subjective, behavioral, and physiological assessments. The use of WIoT technologies that capture in-the-moment experiences and contexts, such as the Oura ring and Samsung Gear Sport smartwatch, has been shown to improve the ecological validity of mental health assessments [58,59]. Thus, a goal of our prospective study is to use a multimodal assessment method that combines data from emerging WIoT technologies and personal chronicles in a daily activity logging framework (ie, the Personicle app) [60,61], to better understand the unique contexts and factors of stress and emotional well-being, as well as the risks and development of psychopathologies among young adults. More specifically, this study aims to investigate daily factors (ie, stressors and activities) and their relationship with the psychopathologies and daily emotions of college students. Ultimately, we believe that our study will help with developing personalized models that can be used to monitor, predict, and treat mental health and well-being issues among emerging adults. Specifically, we test the following big-picture research question: is it possible to build personalized predictive models of mental health for individuals? For example, sleep disturbances and poor social interaction skills can be used as factors for predicting increases in depression severity. However, reduced amounts of physical activity and low positive emotionality are other factors that can be used to predict increases in depression severity over time.

We believe that the methods we describe in this protocol paper may allow psychologists to identify the root causes of stress and develop an evidence-based approach for monitoring stress and emotions among adolescents and young adults. Herein, we provide an overview of our prospective study.

## Methods

### Study Design

#### Eligibility Criteria and Recruitment

Our protocol was approved by the institutional review board at the University of California, Irvine (approval number: 2019-5153). We will recruit participants by distributing flyers throughout the college campus community, disseminating related digital content on social media pages (ie, the University of California, Irvine Facebook pages), sending emails to people on the university listservs, and telling members of the teaching faculty to share study information on their class websites. These methods will hopefully yield a broad and representative sample of college students across different disciplines and years of study. Eligible participants include full-time students from the University of California, Irvine aged 18-22 years, and those who own an Android smartphone (ie, must be students’ primary phone) that is compatible with the Personicle app, ecological momentary assessment (EMA) phone-based surveys, and study devices. Participants are ineligible if they are parents, are married, are returning to school after a period of ≥3 years, or are unable to speak/write English fluently. Eligibility will be determined via email and phone screening, which will be conducted prior to laboratory visits. Participants with indications of suicidal ideation or moderate to severe depression during the survey assessments will undergo additional screening, which will be conducted by one of the lead researchers (ie, JB, a clinical psychologist). The lead researcher may decide to withdraw participants from the study to protect participants’ safety and health. This strategy will ensure that participants with mental health concerns (eg, depression) will still have the opportunity to participate in the study.

#### Data Collection Procedures

**Figure 1:**
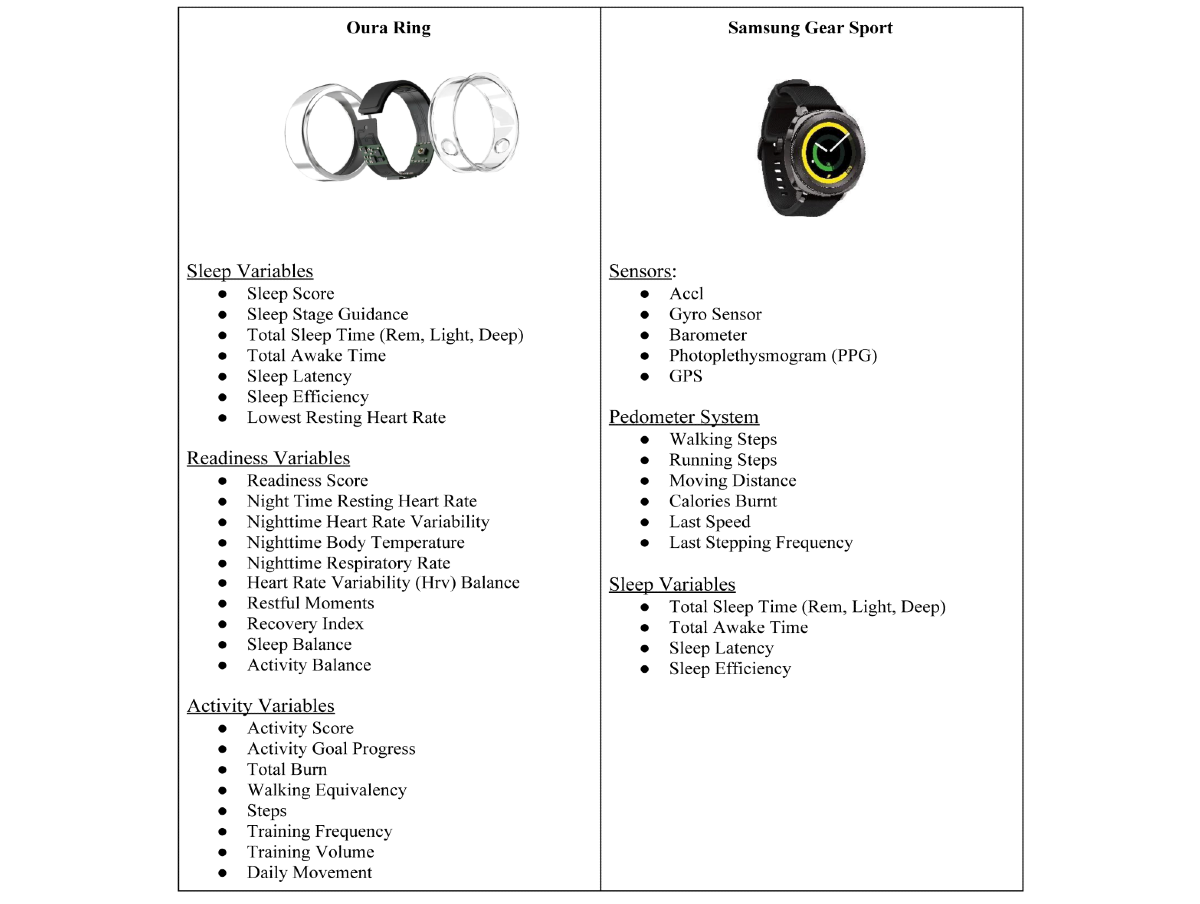
Data that are collected by the Oura ring and the Samsung Gear Sport smartwatch. The Oura ring collects data on sleep, readiness, and activity. The Samsung Gear Sport smartwatch collects data on sleep and activity, by using sensors (ie, a barometer) and a pedometer system.

Our study will involve an in-lab preassessment, followed by a 12-week remote data collection period and an in-lab exit assessment. During the preassessment, participants will fill out a consent form and complete a questionnaire battery that consists of standard psychological and relationship-based measures. Demographic information, including age, year of schooling, gender, ethnicity, and relationship duration, will be collected. After the preassessment, participants will be given noninvasive WIoT devices that assess activity and physiology throughout the day and during sleep, in an effort to capture an accurate depiction of participants’ daily physical habits and health (see Figure 1). Participants will then be asked to download four apps onto their smartphone; one app will be for completing daily surveys on emotion, the second app (ie, Personicle) will collect daily activity data (eg, phone interactions and physical activities), and two others (Oura and Galaxy wearable) in order to use the wearables and track the data. At the end of the 12-week period, participants will complete a battery of questionnaires, which will be similar to their initial assessment battery. At the end of their participation, participants will complete a final, postassessment questionnaire that contains additional questions about technology acceptance, open-ended feedback, and whether they used mental health services during the participation period. Participants will be told to wear their smart devices at all times (ie, if possible) and sync their devices every few days, to ensure that our servers receive the data. Participants will also be informed that they need to maintain a survey completion rate of at least 80%. This percentage was chosen based on previous research studies that required a similar completion rate [62,63]. To maintain high adherence rates and low attrition rates, we will monitor incoming data on a weekly basis to ensure that participants are syncing and wearing their devices and completing the daily survey. Our web-based dashboard provides real-time information on the wear time of sensors and the completion status of surveys. Participants will be contacted if they fall below the weekly 80% assessment completion rate. Participants will also be sent reminders via text message, email, or phone call if more than 2-3 days of inactivity per week are detected. Inactivity will be defined as failing to sync the ring or watch, failing to wear the ring or watch, and failing to complete the daily and weekly survey. Reminders will be primarily sent via text message, but if participants do not respond or do not adhere to study procedures, then the research team will send reminders via email and phone call. We will set up a study-specific Gmail account and Google voice account for contacting participants. The reminders will state something to the effect of the following: “Hello, we have noticed that you have not completed the daily survey within the past 2 days. Your survey completion rate is currently at 70%. To ensure you are completing at least 80% of the surveys, please remember to complete the survey every day.” Furthermore, the reminders will be administered on a case-by-case basis, because survey completion is influenced by external factors, such as the survey app not functioning on a certain day or a wearable device being faulty.

#### Mental Health and Well-Being Assessment Battery

At study intake and at regular intervals thereafter (ie, 3 months following intake and every 3 months after that point), participants will complete a mental health and well-being assessment battery. This battery will be identical at each time point (with the exception of additional measures at the follow-up and postassessment), and will contain a variety of validated, gold-standard assessment tools that are used for measuring an array of mental health symptoms that are common in the emerging adult population. The assessments that the participants will complete include (1) the 21-item Beck Depression Inventory-II [64], which measures the severity of the cognitive, affective, behavioral, and physiological symptoms of depression that people experience over 2 weeks; (2) the 6-item anxiety subscale of the Brief Symptom Inventory [65], in which anxiety severity is rated on a 4-point subscale that ranges from 0 (ie, not at all) to 4 (ie, extremely); (3) the 3-item University of California, Los Angeles Loneliness Scale [66], in which loneliness is rated on a Likert scale that ranges from 1 to 3; and (4) the Brief Coping Orientation to Problems Experienced Scale, which is a 28-item questionnaire on coping responses (eg, substance abuse) for stressful events [67]. At the end of the study, participants will complete one final mental health and well-being assessment battery. With regard to the scales in this battery, we will calculate the internal consistency of each measure and compute participants’ total scores for each measure. Multiple assessments of participants’ mental health and well-being data, and identical measures across time intervals will enable us to examine changes in mental health and well-being indicators across the year.

### Wearable Devices

#### Oura Ring

**Figure 2:**
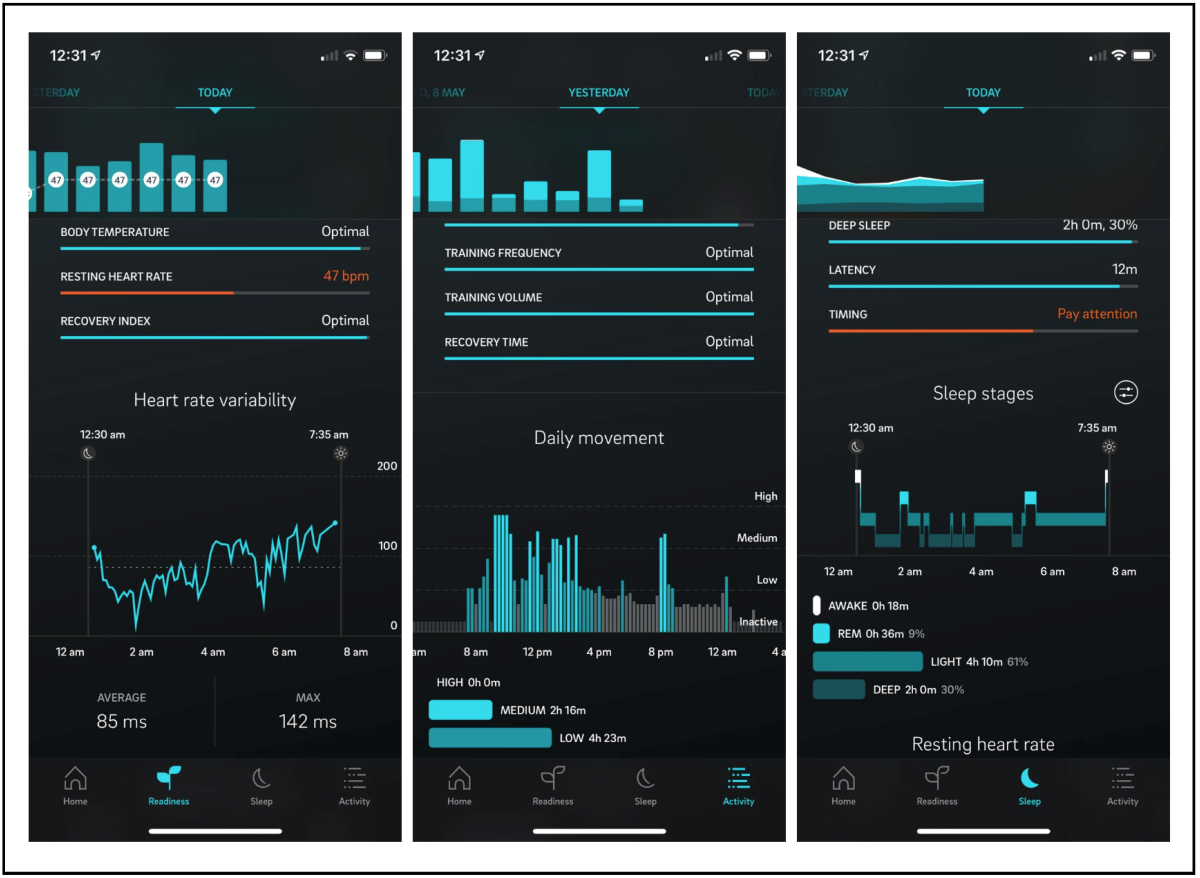
Examples of Oura ring screens that display information on heart rate variability, daily movement, and sleep stages. These screens can be viewed on the Oura phone app.

The Oura ring [68] measures a myriad of physiological variables, which are categorized into three general health areas, as summarized in Figure 1. The Oura ring collects information on sleep, including the time that participants spend in different stages of sleep (ie, the light, deep, and rapid eye movement stages), by detecting and interpreting physiological measures such as heart rate, heart rate variability, and pulse wave variability amplitude [69,70]. The Oura ring will uniquely calculate participants’ activity variables, including energy expenditure and activity level, based on a highly personalized combination of body metrics (ie, height, weight, age, and gender) and 3D accelerometer data. Metabolic equivalents [71] are the Oura ring’s primary unit for measuring energy expenditure. These are taken into consideration when the Oura ring categorizes the intensity of aerobic exercise. Additionally, each participant will be given a personalized activity score on each day. Activity scores reflect participants’ overall activity intensity, activity frequency, and postworkout recovery time. Participants will be given the opportunity to view their data (eg, the weekly trends of each measure) on the Oura app (Figure 2), which participants will install on their phones during the initial assessment session.

#### Samsung Gear Sport Smartwatch

**Figure 3:**
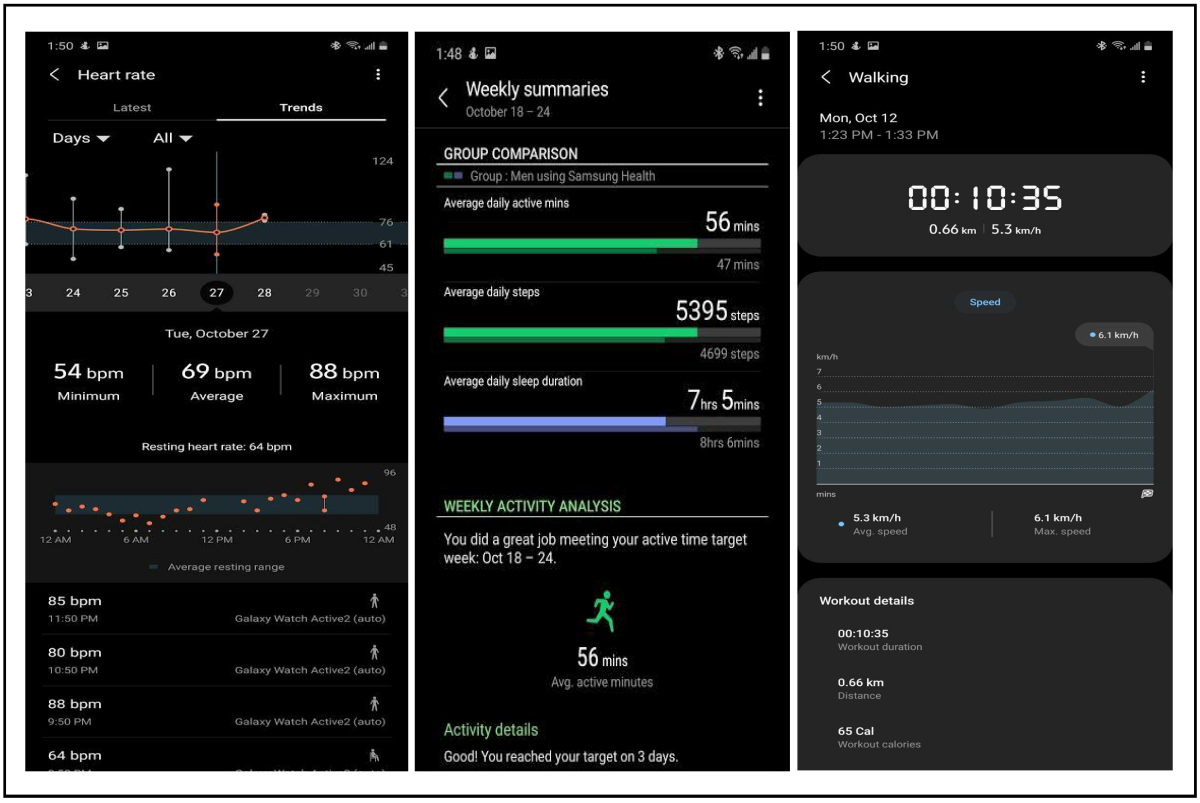
Examples of Samsung Gear Sport activity screens that display cycling information (eg, trends, speed, heart rate, and elevation). These screens can be viewed on the Galaxy Wearable phone app.

In addition to the Oura ring, participants will be given a Samsung Gear Sport smartwatch to wear on a daily basis. The Samsung Gear Sport smartwatch operates on the open-source Tizen Operating System, includes open software development kits, and provides open access to raw signals [72]. The smartwatch’s sensors measure vital signs and signals, such as photoplethysmogram signals, heart rate, heart rate variability, and respiration rate. This allows the smartwatch to assess stress, activity levels, and sleep (see Figures 1 and 3). Furthermore, our research team has developed an app that can be used on this watch. The app extracts raw signals (eg, photoplethysmogram and proper acceleration signals) from the watch’s sensors, which allows us to conduct elaborate biosignal processing and machine learning analyses on data, and to assess complicated phenomena, such as stress. Similar to the Oura ring, the Samsung Gear Sport smartwatch uses a variety of sensors to quantify different activity measures, in an effort to inform wearers of their physical health habits (Figure 1). The watch places a heavy emphasis on exercise and activity metrics, and unlike the Oura ring, the watch uses a gyrosensor and alti-barometer to assess environmental factors, such as altitude and step incline, for calculating variables such as the number of calories burned and moving distance. When paired with the Oura ring, the Samsung Gear Sport smartwatch provides a sensory system that yields an all-encompassing insight into the exercise and activity habits of the participants.

### Personicle App

**Figure 4:**
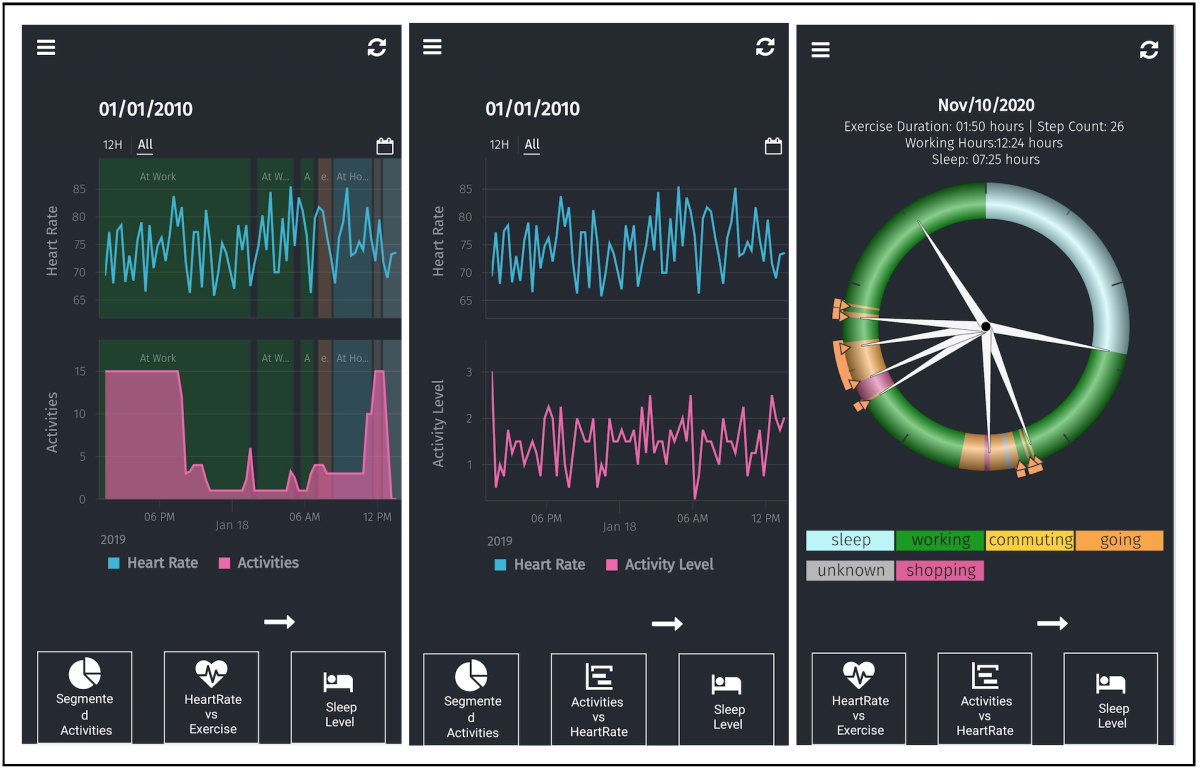
Examples of screens that are shown on the Personicle phone app. These screens display information on physical activity (eg, heart rate) and daily activities (eg, home events).

Personicle is a multimodal personal chronicle of daily activity that automatically integrates heterogeneous sensory data from the IoT (eg, accelerometer, gyroscope, altimeter, GPS, light sensor, and temperature sensor) with contextual, social, and environmental information to create a chronicle of life events (ie, activities and biomarkers) [60,61]. For the purpose of our study, the Personicle app will be used to assess the three following main event categories: activity-related (eg, walking and socializing), health-related (eg, high heart rate variability), and context-related (eg, stressful workplace, parents’ house and friend’s house) events (see Figure 4). To identify daily activities, we built a common daily activity model by identifying the global unique properties of each individual event. Specifically, we used a common event modeling approach to analyze the physical (eg, event occurrence time stamps and intervals), logical (eg, temporal domain), and relative (eg, temporal relationships to other events) relationships between each aspect and an event. We incorporated these general aspects into the categories of our modeling attributes and modified the physical, logical, and relative components to match those of daily activities. We developed an event mining system to identify temporal associations among events, which allowed us to build personalized models [73]. For instance, to understand an individual’s social behavior, we must examine their locations (eg, the amount of time an individual spends at various places, such as a friend’s home or parents’ home). We also used our event mining system to identify linkages between activities (eg, going out for ice cream) and behaviors (eg, driving to friend’s house), which also contribute to building an individual’s personalized model.

### Daily and Weekly Assessments

**Figure 5:**
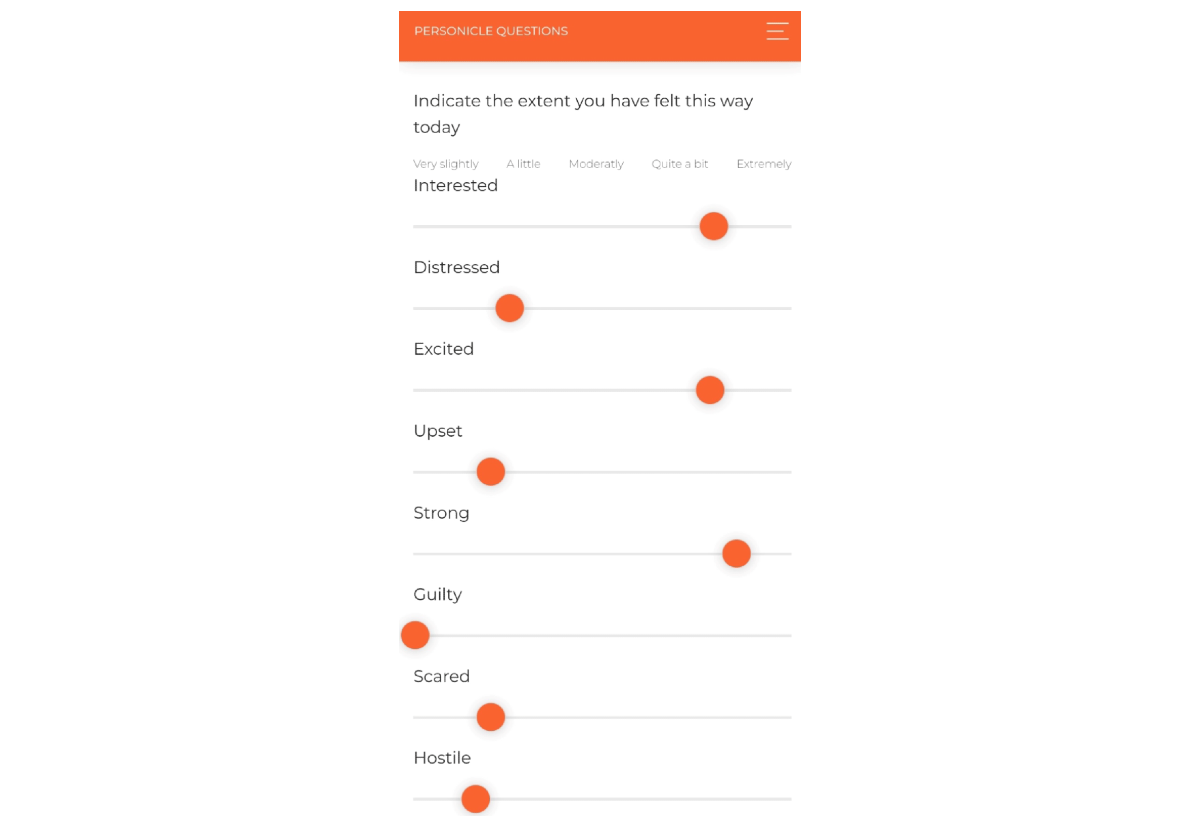
An example of the daily assessment surveys that participants are instructed to complete every evening.

Participants' daily moods will be assessed by using the Positive and Negative Affect Schedule [74], which is a validated measure for assessing both positive and negative affect (ie, inspired, excited, distressed, and upset). The Positive and Negative Affect Schedule is presented as a slide scale with indicators at the top ranging from “very slightly” (0) to “extremely” (100). Emotion assessments will be evaluated with one of the phone apps that participants will install onto their smartphones (see Figure 5). Participants will also answer two open-ended response questions once a week, to provide additional context for their subjective experiences (ie, “Please write about your high points and low points this week. Please try to be as detailed as possible” and “Please rate how you felt about your week”).

### Data Analytic Plan

Since our pilot study aims to assess the feasibility of using an extensive multimodal approach for understanding the holistic aspects of stress and well-being, our analyses are exploratory in nature. Furthermore, our sample size is small. Thus, we will take into account the adherence rate and the amount of collected data during the assessment period. We will contact participants throughout the data collection process in order to maintain an estimated adherence rate of 80% and reduce the amount of missing data. However, missing data will be reviewed and imputed via full-information maximum likelihood estimation, based on the assessments that participants have completed on other days. Since the questionnaire will be conducted at multiple time points throughout the study, we intend to examine whether there were changes in students’ assessments of depression, loneliness, and well-being. More specifically, daily mood assessments will be based on participants’ daily surveys; and daily physiology, sleep, and activity assessments will be based on the data collected from wearable devices. The wearables will allow us to assess physiology and activity at different time points throughout the day. To make these data comparable with those of our daily assessments (ie, assessments that are only conducted once during the day), we intend to aggregate these data to obtain a single value for each day. To examine associations between intensive longitudinal assessment data (ie, data that are collected daily over several months, including daily reported mood and sleep), we will conduct multilevel modeling analyses [75] to account for the multiple assessments of each individual. Time series analysis and dynamic multilevel modeling techniques are common approaches to analyzing intensive longitudinal data and examining temporal associations and trajectories between constructs of interest [76]. However, given our intended sample size of 20 participants, our study design may be underpowered in terms of detecting an effect.

A central aim of our study is to examine the links among daily variables (eg, sleep, physiology, emotion, and Personicle data) and long-term mental health and well-being data. In order to achieve this aim, we will use data reduction strategies. For instance, we are collecting data on a broad battery of mental health and well-being scales in order to obtain a comprehensive assessment of participants’ psychological functioning. By conducting an exploratory factor analysis, we will be able to examine whether these measures load onto underlying factors (eg, mental health, internalizing, and externalizing symptoms), and reduce the number of analyses we need to conduct. Furthermore, we will use multilevel modeling techniques to examine whether repeated measures data (eg, sleep, emotion, physiology) can be used to predict changes in mental health and well-being data.

In addition to the use of multilevel modeling approaches for examining associations among assessments over time at the within-person level, we intend to use idiographic and network analytic approaches for examining personalized models of mental health. Researchers have long used unified structural equation modeling (SEM) and dynamic SEM to create idiographic personalized models of personality and mental health [77,78]. Unified SEM is particularly helpful because it uses the group iterative multiple model estimation approach to identify components of symptoms and associations among constructs within an individual. These data can be used to examine similarities across the sample [79,80]. Furthermore, network analyses that are conducted by using a multivariate time series analysis approach may also offer better insight into key psychopathology symptoms that an individual experiences [81,82]. Despite the small sample size and the possibility of participants withdrawing from the study, we will have numerous observations for each individual over the course of 12 months (ie, an estimated 3000-5675 observations in total). Thus, we will use multilevel modeling to assess the relationships among an individual's daily emotions, activities, and sleep patterns at the within-person level. We may be able to examine individual cases, wherein the relationships among an individual’s emotions, activity, and sleep patterns may differ from those of another individual. For example, daily negative affect and poor sleep quality may be strongly related for one participant, whereas daily positive affect and increased activity levels may be strongly related for another participant.

## Results

We expect 20 participants to complete the study. We recruited an initial cohort of participants between January 2020 and March 2020 (N=10), and they are expected to complete the 12-week data collection period and the following 9-month extension period. Due to the unexpected COVID-19 pandemic, we will make subsequent adjustments and additions to our study. We will change the consent form process and initial survey assessments so that they can be administered and completed via internet-based methods. We will also develop a procedure that will enable us to mail study devices to participants’ homes. A research assistant will help participants set up the devices via video call. Furthermore, COVID-19–related questionnaire measures will be added to the battery questionnaire and daily survey, to assess the pandemic’s psychological impact on participants. Due to the COVID-19 pandemic, participants will be offered to participate in an extension of the study, which will prolong their participation period by up to 9 months. During this extension period, participants will continue to undergo the same study procedures and complete follow-up assessment questionnaires every 3 months. To reduce attrition rates and increase adherence rates, participants’ compensation will be increased for the additional months of participation. Research assistants will send participants encouraging reminders about the potential importance of our study, in order to underscore the fact that their participation is an important contribution to research. In doing so, we hope to incentivize participant adherence. Thus, we aimed to recruit a second cohort (N=10) during the months of June and July 2020. This cohort will be expected to participate in our study until the end of the year. Given that we will be analyzing participant data over the span of a year, there may be certain time points in which participants may encounter challenges to completing the daily assessments of the study. We recognize that there may be periods of time wherein we miss large amounts of data. We hope that by closely focusing on periods of time (ie, over a 3-month span) and specific events (ie, the start of the shelter-in-place during the pandemic, and before and after the US presidential election), we will be able to examine participant data over long periods of time and reduce the amount of missing data.

The use of wearable sensors for 12 months comes with the inevitable issue of missing data. Our group has devised a multitier strategy to mitigate the amount of missing sensory data. Our study uses a multimodal data collection process that involves the long-term aggregation of data from several sources. Even though several challenges arise when implementing and managing such a process, we believe that a by-product of this process is inherent data resiliency, which will enable us to apply data imputation techniques for minimizing the amount missing data. The main reasons for missing data include the following: (1) a user is unable or not willing to wear the device for several periods of time; (2) the device runs out of battery power for a period of time; (3) device failure occurs; or (4) the device is momentarily detached from the body. These may result different forms of missing data, as follows: (1) missing completely at random data, which include data that are missed due to sensor failure; (2) missing at random data, which include data that are missed because the device is detached from the body (eg, times when the device is charging); and (3) not missing at random data, which include data that are missed when a user removes the device (eg, before smoking) to hide an activity’s effect on vital signs. We will use well-accepted data imputation techniques, such as deletion methods (eg, listwise or pairwise deletion), multiple imputation methods, model-based methods (eg, direct maximum likelihood estimation), machine learning–based methods, and multisource methods, based on the type of missing data. Our criteria for selecting data imputation techniques include (1) unbiased parameter estimates; (2) acceptable estimates of variability (ie, correct standard errors); and (3) the highest statistical power. We will also use a technique that was recently proposed by our group; this involves a missing data–resilient, decision-making, personalized approach for assessing health care IoT devices [83]. This method has been validated in an 8-month continuous maternity care project [84].

In summary, we will take advantage of the multimodality nature of two separate wearables (ie, sensory inputs, personalization, and the redundancy of different signals), to perform advanced data imputation techniques for recovering missing data or mitigating the amount of missing data. Furthermore, a web-based dashboard will assist the research team in the early identification of technical issues during monitoring. The research team will also receive alerts about the occurrence of missing data (eg, users not wearing the sensors for a period of time, the research team not receiving data packets due to internet connection issues, etc).

## Discussion

### Contributions

To our knowledge, ours is one of the first studies to use EMA surveys, wearable smart devices, and a personal event life logging system to record daily moods and events to build a personalized model for predicting changes in the mental health and well-being of college students. A strength of our ongoing study is that we have an immense amount of rich data from participants that have been analyzed on a daily basis over the course of 1 year. Our study is also unique because we collected data before the COVID-19 pandemic and during the pandemic. This provided us with the opportunity to analyze patterns in everyday life during a pandemic. Furthermore, due to the COVID-19 pandemic, students face the challenge of using remote methods to maintain social relationships and complete coursework. This will further exacerbate well-being issues among college students [85,86]. Thus, our study has the ability to examine well-being patterns among college students that use remote learning methods during the pandemic. Additionally, the results of our pilot study will help Personicle become a better open-source IoT app that is available to the public.

### Limitations

We anticipate that our study will have limitations that are similar to those of many other EMA-based studies, such as nonadherence to study procedures and experimental fatigue. These limitations might result in missing data [87]. Furthermore, participant burden may be reflected by the data quality of completion times for daily surveys (ie, taking time to select answers vs carelessly selecting answers), and biased responses (ie, the influence of the research team sending reminders and conducting follow-up examinations). Longitudinal studies may also have unintended effects, such as participants engaging in healthier behaviors when tracking their own emotions and health [88]. Despite these limitations, WIoT devices allow for naturalistic data collection processes that reduce the burden on participants. We also attempted to reduce participant burden by keeping daily assessments brief. Furthermore, participants who fail to meet our criteria for adherence rate (ie, participants with a weekly assessment completion rate of only 10%) will be withdrawn from the study. We will recruit additional participants to achieve our target sample size (ie, N=20), and keep careful records about the replacement of participants. As we are assessing college students over the course of a year, we may be able to include time as a covariate for examining changes in well-being over time (eg, changes in sleep patterns or physical activity over time). Additionally, several of the biggest limiting factors of the Personicle app include its general definitions for activities, its inability to collect data on a wide range of events, and its inability to distinguish specific events from a large segment of events.

Since the existing Personicle system was modified for our specific study, we expect that multiple system updates will be implemented to fix bugs and other issues. Although the refinement of the system may provide us with more accurate data, it might lower the accuracy and interpretability of previously collected data. We expect the need to notify enrolled participants about updating their Personicle app to the most recent version if updates do occur over the course of the study. However, a benefit of the Personicle system is that it allows users to send Personicle system logs (ie, files that have all the data that the app has collected) directly to the server. This will allow the research team to identify missing data or issues.

Another limitation is that our study began in January, with the intention of assessing students over the course of a certain period of time. However, since the COVID-19 pandemic has disrupted the daily lives of many individuals, our research plans had to be adjusted to account for participants’ experiences during a pandemic. To study the impact of the pandemic, we incorporated additional questions into the daily assessments (ie, “Please rate how worried you felt about your health today” and “How worried were you about contracting COVID-19 today”). Participants will answer these questions by using a sliding scale that ranges from 0 (ie, not worried at all) to 100 (ie, extremely worried). Despite these changes, the amount of data that we collected over the course of the year and the COVID-19 pandemic offers potentially interesting insight into individualized experiences and well-being.

### Conclusion

In the context of an individualized approach to understanding mental health and well-being, using WIoT devices and the Personicle app as a multimodal system allows us to conduct root cause analyses and study interrelations among stress, social relationships, technology, and mental health. Our study will provide fundamental contributions to the field of computing, as we investigate a holistic, cybernetic, closed-loop architecture for personalized model generation. Our study will also contribute to psychological science, as we have created an evidence-based approach based on individualized, Personicle-generated feedback for reducing stress and negative emotionality in adolescents and young adults.

## Abbreviations

DSM: Diagnostic and Statistical Manual
EMA: ecological momentary assessment
IoT: Internet of Things
SEM: structural equation modeling
WIoT: wearable Internet of Things
